# Drinking Water in Transition: A Multilevel Cross-sectional Analysis of Sachet Water Consumption in Accra

**DOI:** 10.1371/journal.pone.0067257

**Published:** 2013-06-19

**Authors:** Justin Stoler, John R. Weeks, Richard Appiah Otoo

**Affiliations:** 1 Department of Geography and Regional Studies, University of Miami, Coral Gables, Florida, United States of America; 2 Department of Public Health Sciences, Miller School of Medicine, University of Miami, Miami, Florida, United States of America; 3 Department of Geography, San Diego State University, San Diego, California, United States of America; 4 Ghana Urban Water Limited, Head Office Operations, Accra, Ghana; Chancellor College, University of Malawi, Malawi

## Abstract

Rapid population growth in developing cities often outpaces improvements to drinking water supplies, and sub-Saharan Africa as a region has the highest percentage of urban population without piped water access, a figure that continues to grow. Accra, Ghana, implements a rationing system to distribute limited piped water resources within the city, and privately-vended sachet water–sealed single-use plastic sleeves–has filled an important gap in urban drinking water security. This study utilizes household survey data from 2,814 Ghanaian women to analyze the sociodemographic characteristics of those who resort to sachet water as their primary drinking water source. In multilevel analysis, sachet use is statistically significantly associated with lower overall self-reported health, younger age, and living in a lower-class enumeration area. Sachet use is marginally associated with more days of neighborhood water rationing, and significantly associated with the proportion of vegetated land cover. Cross-level interactions between rationing and proxies for poverty are not associated with sachet consumption after adjusting for individual-level sociodemographic, socioeconomic, health, and environmental factors. These findings are generally consistent with two other recent analyses of sachet water in Accra and may indicate a recent transition of sachet consumption from higher to lower socioeconomic classes. Overall, the allure of sachet water displays substantial heterogeneity in Accra and will be an important consideration in planning for future drinking water demand throughout West Africa.

## Introduction

Population growth in the developing world continues to put a strain on drinking water supplies, even amid declining fertility. As of 2010, approximately 884 million people–over a third of whom live in sub-Saharan Africa–still did not have access to an improved drinking water source [1]. Sub-Saharan Africa is the only region not on track to meet the Millennium Development Goal target of halving the proportion of the population without sustainable access to safe drinking water [1]; the percentage of individuals with access to piped water in the dwelling, yard, or plot stagnated from 1990–2010 and fell in urban areas as urban populations grew by over 30% [2], [3]. Despite international efforts to extend access, morbidity and mortality attributable to inadequate water and sanitation remain high, particularly for children under five [4]. In Ghana, rapid urbanization continues to erode the government’s ability to provide municipal water to its urban centers. The percentage of the urban population with access to an improved water source increased from 84% in 1990 to 90% in 2008, yet the percentage with access to piped water decreased steadily from 41% to 30% [1].

In Accra, Ghana’s coastal capital, drinking water shortages are not driven by lack of surface or ground water *per se*, but are historically attributable to poor governance and improper water resource management [5]. Population growth and urban water mismanagement have resulted in water demand in Accra that far exceeds the water production capabilities of the two local water treatment plants. Ghana Urban Water Ltd. (GUWL; a subsidiary of the Ghana Water Company Ltd.) has instituted a rationing program for water distribution within city limits [6], and by one estimate 75% of Accra lacks 24-hour water access while another 10% has no access at all [7]. Municipal water rationing varies both geographically and socioeconomically by neighborhood in Accra [8], and these service gaps have been linked to the recent ubiquity of packaged “sachet water” sold in sealed 500 ml plastic sleeves. Over the last five years, sachet water has become an important primary drinking water source for the urban poor, and may even confer a health benefit when sachets replace the consumption of improperly stored water in the home [8]. As piped water access in Accra continues to decline, it remains unclear whether sachet water is strictly a phenomenon of the poor, or if sachets are being consumed by a more socioeconomically diverse population and with similar protective health effects.

The most recent Joint Monitoring Report from WHO/UNICEF [3] indicates that urban access to piped drinking water is on the decline throughout sub-Saharan Africa, a trend previously observed over three decades in East Africa [9], but the report attributes these service losses solely to population growth. International development agencies continue to ignore West Africa’s transition to sachet water with no mention of it in several recent regional reports [1], [3], [10]–[12]. WHO’s recent report on household water treatment and storage [13] discusses routine and emergency distribution of flocculent-disinfectant sachets for household use, but there is no discussion of pre-treated sachet water in the form addressed here. Because urban drinking water options have evolved over just the last few years, a fuller understanding of West Africa’s urban drinking water patterns is crucial for catching up on missed Millennium Development Goals for sustainable drinking water and sanitation in the region.

This study utilizes survey data from the Women’s Health Study of Accra (WHSA) to analyze the socioeconomic and geographic patterns associated with sachet water consumption and the effect of water rationing on sachet consumption. We present data on primary drinking water sources and socioeconomic characteristics for a sample of 2,814 women in Accra. Because GUWL water rationing is managed by local water districts, we expect that a household’s drinking water options are influenced by the degree of neighborhood rationing after accounting for individual differences. We specifically test three hypotheses: (1) that residents of lower socioeconomic status are more likely to consume sachet water as their primary water source, (2) that residents enduring a greater number of days of water rationing in the neighborhood are more likely to consume sachets, and (3) that the interaction of higher rationing and lower socioeconomic status will produce the highest rates of sachet use. In light of sachet water’s massive appeal throughout West Africa, we comment on the sustainability of sachets as an urban drinking water solution, as well as how the phenomenon might better inform water provision projects in the region.

## Methods

### Study Population

Accra is typical of many developing urban areas in West Africa both for its progress in health care delivery and its overall epidemiologic transition. The Accra metropolitan area, with its southern border on the Gulf of Guinea coast, extends about 11 km north just beyond the University of Ghana at Legon, and is roughly 20 km east to west. The metropolitan area contained 1.66 million people and 373,540 households according to the March 2000 Accra census, and is approaching 2.4 million people as of the 2010 census, while still growing at 3% annually [2]. Local governance of this population rests with the Accra Metropolitan Assembly, and logistical coordination with this single administrative body facilitated implementation of the study instrument.

### Study Instrument

**Figure 1:**
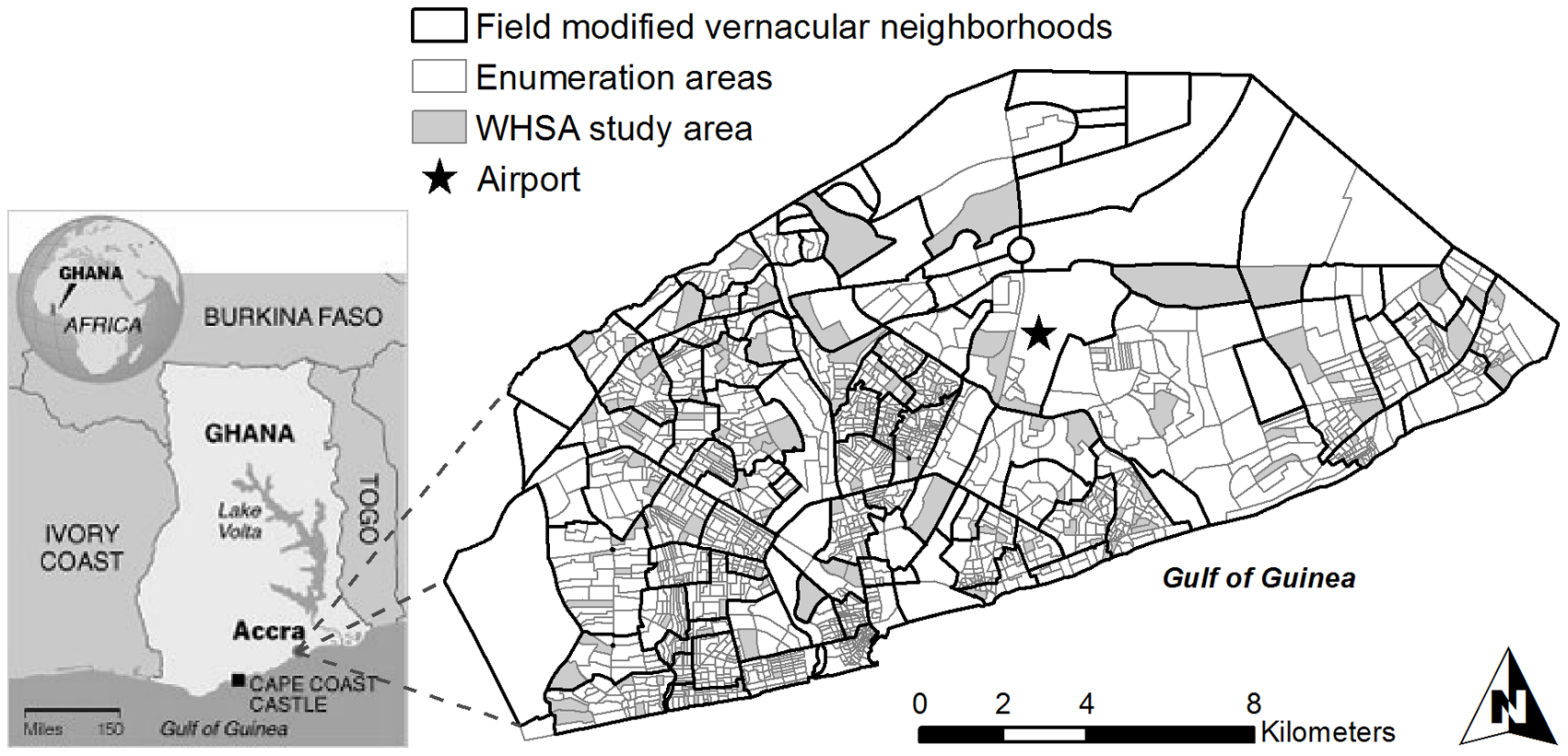
Digital layer of the study site in Accra, Ghana, depicting WHSA-II study sample with enumeration area (EA) and field modified vernacular neighborhood (FMVN) levels of analysis.

The WHSA is a community-based longitudinal population study conducted in Accra by the Harvard School of Public Health, the University of Ghana at Legon, and San Diego State University. The first round of the WHSA was conducted in 2003, with a follow-up survey in 2008–2009. The survey provides a comprehensive snapshot of health, disease, and related risk factors in each study year among a representative sample of women ≥18 years of age in the Accra metropolitan area [14], [15]. Participants were originally selected through a two-stage cluster probability sample stratified by socioeconomic status (SES) quartiles, as determined by the 2000 census, and by four age groups. The sample was drawn from a population-weighted sample of 200 of Accra’s 1,731 enumeration areas (EAs), and study women were selected according to the probability of being in one of 16 cells formed by the stratification of SES and age group [14]. To improve our understanding of neighborhood context, EAs are aggregated into 108 neighborhood units called *field modified vernacular neighborhoods* (FMVN) [16], which are field-validated configurations of previously-defined *vernacular neighborhoods* [17]. The study sample and spatial hierarchy are depicted in Figure 1.

The study interviewed 3,172 women in 2003, but due to significant challenges tracking women between surveys (primarily the lack of a formal address system to trace those who moved, and also Institutional Review Board prohibition of collecting original household location data via Global Positioning System receivers in 2003), 39% of women were lost to follow-up in 2009, in addition to 5% that were deceased. The 2008–2009 round of the WHSA (henceforth referred to as WHSA-II) interviewed 2,814 women, 995 of whom were replacements–matched by age and enumeration area–for those lost to follow-up. This paper explores the recent sachet water phenomenon, which was not an important factor in 2003 when 97.5% of WHSA respondents reported piped water access (either in or outside the home) as their primary drinking water source. In order to analyze the new dynamics of sachet water using the broadest sample possible, we restrict ourselves to a cross-sectional application using only the WHSA-II data set with an explicit acknowledgment of limitations on interpreting causality from the results.

All WHSA-II participants provided written informed consent; literate women provided their personal signature, while illiterate women provided an ink fingerprint in lieu of a signature. Institutional Review Board approval for all consent procedures, data collection, and analysis was granted by the respective boards of Harvard University, University of Ghana-Legon, and San Diego State University (SDSU).

### Statistical Analyses

To test hypothesis 1, we use *Χ* ^2^ and *F* tests to compare socioeconomic and demographic characteristics between sachet drinkers and those using all other drinking water sources. To test hypothesis 2, we implement a series of iterative multilevel logistic regression models to separate compositional and contextual effects associated with sachet consumption as a primary drinking water source, with neighborhood factors treated as the exposures of interest. We use exploratory forward and backward stepwise regression models to advise the introduction of independent measures in the multilevel model-building process. The full model parsimoniously maximizes higher-level covariance parameters while minimizing the model fit statistic (−2 restricted log pseudo-likelihood). To test hypothesis 3, we introduce cross-level interactions with rationing into the full model, as well as individual-level interactions for other significant measures. We implement a random intercept model using the GLIMMIX procedure in SAS 9.2 with parameters fitted to the FMVN, EA, and/or woman as described below.

The theoretical underpinnings of measuring health and poverty through socioeconomic indicators have long been established in the social epidemiology and international development literature [18]–[21]. In this analysis, the individual effects modeled include respondent demographics such as age, ethnicity, and education; household characteristics such as dwelling type, and access to toilets and waste disposal services; and an individual-level measure of water pipe density within 500 m of the household (generated from a kernel density surface estimation of neighborhood water pipe penetration). We control for SES quartiles at the EA level, then introduce neighborhood, i.e. FMVN-level, factors that have been previously linked to adverse health outcomes: infrastructure conditions such as pipe density and days of water rationing [8], the proportion of vegetated land cover as a proxy for socioeconomic status [22], and a slum index and housing quality index constructed from 2000 census data to infer socioeconomic status at a finer level [16], [22]. The proportion of vegetated land cover was summarized for each FMVN and EA from a classification of a high spatial resolution (2.4 m) QuickBird image captured in 2010. GUWL rationing metrics were recorded in July 2009 and are drawn from schedules of days per week of water service as implemented by local water districts in 2009. Rationing data are managed by GUWL as a GIS point layer and mapped as a surface of Theissen polygons; mean rationing values are summarized at the FMVN scale by an area-weighted algorithm. All FMVN-level measures were also calculated (or disaggregated) at the EA-level for comparison purposes, and the multilevel models were computed as 3-level (FMVN, EA, individual) and 2-level (EA, individual) models.

## Results

Table 1 summarizes the individual characteristics of women from the WHSA-II interviews. Among 2,814 women in the study, 6.8% named sachet water as their primary drinking water source. The demographic profile of these women, compared with non-sachet users, was about 3 years younger on average, disproportionately more likely to live in an EA that ranks in the lowest SES quartile, and more likely to be any ethnicity other than Ga, particularly ethnic groups such as Mole-Dagbani that are grouped into the *other* category. There were no statistically significant differences observed between sachet drinkers and their counterparts in education, though sachet drinkers were significantly more likely to report lower levels of overall health. A profile of sachet consumers as younger, lower-income ethnic minorities begins to emerge, but additional socioeconomic details in Table 1 offer conflicting results.

**Table 1:** Women’s individual characteristics from the WHSA-II.

	Sachet Water		Other Water Source	
Characteristic	Freq.	% or mean (95% CI)	Freq.	% or mean (95% CI)
*Individual characteristics (*%, *n = 2,814)*	190	6.8	2,624	93.2
Age (years) *		43.5 (41.1–46.0)		46.5 (45.8–47.2)
Major Ethnic Group (%) ***				
Akan	65	34.2	850	32.4
Ewe	33	17.4	358	13.6
Ga	53	27.9	1,085	41.3
Other	39	20.5	331	12.6
Education (%) ∼				
None, other, religious	43	22.8	568	21.8
Primary	29	15.3	306	11.7
Middle	57	30.2	1,043	40.0
Secondary	36	19.0	435	16.7
Higher	24	12.7	255	9.8
Self-reported overall health (%) ***				
Excellent	5	2.6	319	12.3
Very good	33	17.5	650	25.0
Good	108	57.1	1,238	47.6
Fair or poor	43	22.8	396	15.2
Socioeconomic status quartile of EA ***				
Lower class	78	41.1	707	26.9
Lower middle class	36	18.9	605	23.1
Upper middle class	36	18.9	681	26.0
Higher class	40	21.1	631	24.0
Type of dwelling (%)				
House, semi-detached, flat	63	33.2	820	31.3
Compound house	125	65.8	1772	67.6
Hut, tent, kiosk, business, other	2	1.1	28	1.1
Number of rooms in dwelling		2.5 (2.3–2.8)		2.5 (2.4–2.6)
Solid waste disposal (%) **				
Collection service	83	43.7	894	34.1
Public dump	88	46.3	1,516	57.8
Burnt, buried, dumped elsewhere, other	19	10.0	214	8.2
Liquid waste disposal (%) ∼				
Sewage system	22	11.6	438	16.7
Thrown in street, gutter, compound, other	168	88.4	2,186	83.3
Type of toilet access (%)				
WC or another house	73	38.4	978	37.3
KVIP or public toilet	88	46.3	1268	48.3
Pit latrine, bucket/pan, other, none	29	15.3	378	14.4
Type of bathing facility (%)				
Own bathroom	75	39.5	873	33.3
Shared with other households	109	57.4	1,635	62.3
Cubicle, open space, other	6	3.2	116	4.4
Wealth score ∼		0.11 (−0.02–0.24)		−0.01 (−0.05–0.03)
Pipe density (mm/km^2^) within 500 m *		819 (732–906)		920 (892–947)

∼*p*<0.10;

* *p*<0.05;

** *p*<0.01;

*** *p*<0.001.

Note: *p*-values for categorical measures are from *Χ* ^2^ test; *p-*values for continuous measure are from Welch *F* test of equality of means to account for variance heterogeneity.

Women relying on sachet water were less likely to be connected to a sewage system for liquid waste disposal (11.6% vs. 16.7%), an observation that is consistent with the “low income” profile, but was not statistically significant. Conversely, sachet drinkers were significantly more likely to report using a collection service for solid waste disposal (43.7%) than those drinking from other water sources (34.1%), yet access to a waste collection service is generally associated with higher-income populations in Accra. Solid waste disposal was the sole household socioeconomic variable that yielded statistically significant differences between sachet drinkers and the rest of the study population (*p* = 0.009). There were other non-significant hints of better amenities among sachet drinkers in Table 1: sachet drinkers were more likely than their counterparts to use their own bathroom for bathing (39.5% vs. 33.3%), and rated higher on a wealth score of durable goods ownership (mean 0.11 vs. -.01), a difference that translates to the 62^nd^ vs. 59^th^ percentile. There were no significant differences in dwelling type, rooms per dwelling, type of bathing facility, and toilet access. All of these variables except toilet access have previously differentiated sachet drinkers as being of lower means than other women in Accra [8]. The density of GUWL pipe infrastructure was calculated within varying buffers from each household, and sachet users had lower pipe density values within 500 m than non-sachet users (819 vs. 920 mm/km^2^, *p* = 0.03).

**Figure 2:**
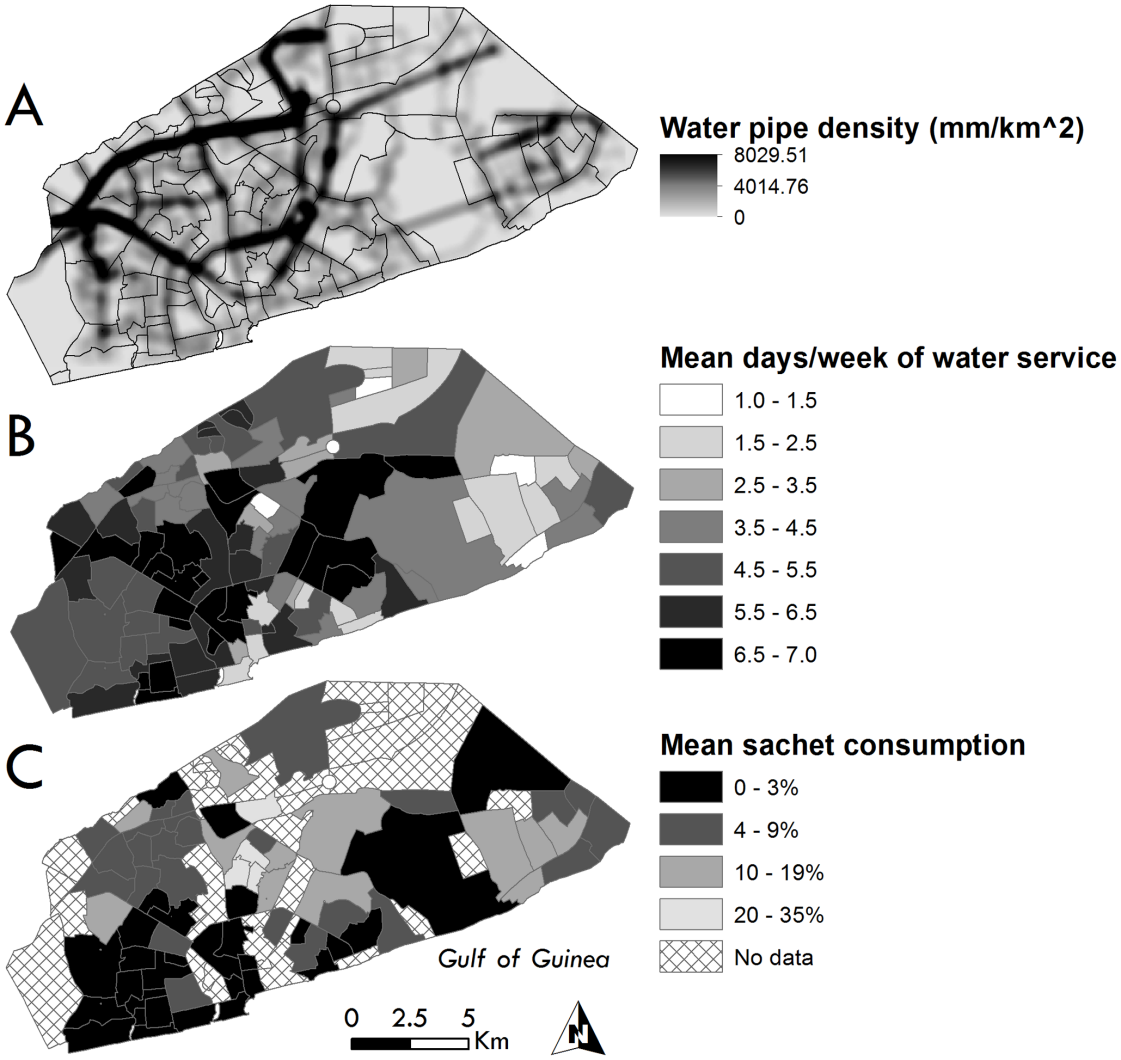
Accra metropolitan area neighborhoods (FMVN) depicting (A) mean density of 2009 GUWL water pipe infrastructure (mm/km2) using a 500 m kernel, (B) mean days per week of running water service according to the 2009 GUWL rationing regime, and (C) smoothed mean percentage of women reporting sachet water as the primary drinking water source for 71 FMVN sampled in the 2008–2009 WHSA-II.

Table 2 summarizes the overall mean FMVN-level characteristics and Pearson’s correlations for 71 neighborhoods that encompass the WHSA-II study population, and selected characteristics are mapped in Figure 2. The neighborhood mean GUWL pipe density was 1,074 mm/km^2^, a figure higher than the household means because major pipelines tend to co-locate with major roads that are not necessarily residential centers; the kernel density surface of water pipe density is depicted in Figure 2A. Each FMVN averaged about 5 days per week of running water service due to the GUWL rationing regime as shown in Figure 2B. Neighborhood pipe density was weakly correlated with days of running water, implying that water service was truly more dependent on a local water district’s rationing policy than on local infrastructure capacity. The raw neighborhood average for sachets as the primary drinking water source was 7.5%; to account for small sample denominators in several neighborhoods, we applied an empirical Bayes smoothing algorithm and mapped the smoothed sachet rates in Figure 2C (smoothed mean = 6.6%). As shown in Table 2, neither neighborhood-level sachet metric was correlated with any of the other neighborhood measures. Three additional FMVN-level measures of socioeconomic variation were considered: the proportion of vegetated land cover (mean 19.86%, SE 2.00), a slum index (2.00, 0.04), and a housing quality index (2.44, 0.07). While these socioeconomic proxies were statistically significantly correlated with each other, none yielded significant correlations with the water-related neighborhood-level measures in Table 2.

**Table 2:** Characteristics and Pearson’s correlations of 71 Field Modified Vernacular Neighborhoods (FMVN) comprising the WHSA-II study population.

Characteristic	Mean	SE	Range	Pearson’s Correlation						
				WPD	H2O	SAC1	SAC2	VEG	SI	HQI
GUWL water pipe density (mm/km^2^) (WPD)	1074	73	207–3018	1.00						
Days per week of running water (no rationing) (H2O)	4.92	0.20	1–7	0.16	1.00					
Sachets as primary water source (raw %) (SAC1)	7.53	1.81	0–100	0.16	0.09	1.00				
Sachets as primary water source (smoothed %) (SAC2)	6.59	0.75	0.62–35.57	0.09	−0.04	0.73*	1.00			
Vegetated land cover in 2010 (%) (VEG)	19.86	2.00	0–83.38	−0.14	0.05	0.12	0.12	1.00		
Slum index (SI)	2.00	0.04	1.13–2.57	−0.16	−0.07	−0.04	0.00	−0.65*	1.00	
Housing quality index (HQI)	2.44	0.07	1.58–3.51	0.06	0.01	0.04	0.02	0.79*	−0.92*	1.00

* *p*<0.01 (2-tailed).

Table 3 contains the empty and full 2-level multilevel models. The empty model is the simplest form of the multilevel model (no covariates), and we estimated this form as a baseline for assessing the change in higher-level variance as we added independent measures. We began by fitting 3-level models with the FMVN atop the spatial hierarchy, but the introduction of interaction terms resulted in unstable models with an unexpected inflation of variance. This lack of fit may indicate that the FMVN scale is not optimal for assessing the role of rationing, despite a significant relationship between rationing and sachet use in these models (not shown). In lieu of “overfitting the data,” the model was simplified to two levels with neighborhood parameters fitted at the EA level.

**Table 3:** Beta coefficients (SE) for random intercept models assessing the association between rationing and sachet use among Accra women.

	Model 1		Model 2		Model 3		Model 4		Model 5	
Characteristic	Empty (no covariates)		Full model of EA- and individual-level factors		Full model with level-2 interactions		Full model with level-2 and cross-level interactions		Full model with level-1 interactions	
Intercept	−3.077***	(0.149)	−5.133***	(0.825)	−5.830***	(0.942)	−6.061***	(1.138)	−6.678***	(1.807)
*Interactions: Level-2 (EA)*										
Rationing×Vegetation					−1.420	(0.875)	−1.648∼	(0.889)		
Rationing×Upper middle class SES					−0.404∼	(0.241)	−0.389	(0.243)		
Rationing×Lower middle class SES					−0.072	(0.230)	−0.047	(0.238)		
Rationing×Lower class SES					−0.176	(0.230)	−0.109	(0.236)		
*Interactions: Cross-level*										
Rationing×OH Very good							−0.106	(0.216)		
Rationing×OH Good							−0.150	(0.205)		
Rationing×OH Fair or poor							−0.151	(0.217)		
Rationing×wealth score							0.084∼	(0.050)		
Upper middle class SES×wealth score							0.156	(0.218)		
Lower middle class SES×wealth score							−0.405	(0.309)		
Lower class SES×wealth score							0.627**	(0.231)		
*Interactions: Level-1 (Individual)*										
Wealth score×OH Very good									0.213	(0.634)
Wealth score×OH Good									0.612	(0.606)
Wealth score×OH Fair or poor									0.896	(0.628)
Age×OH Very good									−0.035∼	(0.019)
Age×OH Good									−0.007	(0.007)
Age×OH Fair or poor									−0.029**	(0.011)
*Level-1 (Individual) variables*										
Self-reported overall health										
Excellent†										
Very good			1.264*	(0.502)	1.259*	(0.502)	1.593∼	(0.822)	2.955∼	(1.745)
Good			1.853***	(0.483)	1.855***	(0.484)	2.312**	(0.796)	2.610	(1.646)
Fair or poor			2.151***	(0.516)	2.139***	(0.517)	2.633**	(0.833)	3.988*	(1.711)
Solid waste disposal										
Burnt, buried, dumped elsewhere, other†										
Public dump			−0.355	(0.303)	−0.326	(0.304)	−0.391	(0.305)	−0.428	(0.307)
Collection service			0.104	(0.323)	0.109	(0.325)	0.078	(0.327)	0.038	(0.327)
Liquid waste disposal										
Sewage system†										
Thrown in street, gutter, compound, other			0.592∼	(0.333)	0.578∼	(0.334)	0.497	(0.344)	0.484	(0.354)
Major ethnic group										
Other										
Ewe			0.024	(0.300)	0.015	(0.310)	0.004	(0.304)	−0.019	(0.309)
Ga			−0.255	(0.290)	−0.275	(0.291)	−0.312	(0.293)	−0.292	(0.296)
Akan			0.012	(0.269)	−0.007	(0.270)	−0.011	(0.273)	0.004	(0.275)
Age (years)			−0.016**	(0.005)	−0.016**	(0.005)	−0.015**	(0.006)	–	(–)
Wealth score			0.380**	(0.125)	0.392**	(0.126)	–	(–)	–	(–)
*Level-2 (EA) variables*										
SES quartile										
Upper class†										
Upper middle class			−0.057	(0.449)	0.827	(0.645)	0.718	(0.659)	0.720	(0.664)
Lower middle class			0.523	(0.482)	0.674	(0.695)	0.431	(0.712)	0.381	(0.717)
Lower class			1.354**	(0.489)	1.776*	(0.712)	1.703*	(0.724)	1.704*	(0.728)
% Vegetated land cover			2.069	(1.290)	4.881*	(1.964)	5.452**	(1.988)	5.464**	(2.010)
Days of rationing			0.087	(0.069)	0.395∼	(0.233)	0.514∼	(0.311)	0.531∼	(0.312)
*Random Effects*										
Level-2 variance (EA)	2.040	(0.368)	1.691	(0.349)	1.636	(0.354)	1.668	(0.361)	1.711	(0.368)
Δ (%) in level-2 variance	–		−17.1%		−19.8%		−18.2%		−16.1%	
−2 res log pseudo-likelihood	15,945.99		15,791.81		15,905.96		16,083.04		16,285.13	
Generalized chi-square	1,427.79		1,429.90		1,483.29		1,504.83		1,527.98	

† Reference Category;

∼ *p*<0.10;

* *p*<0.05;

** *p*<0.01;

*** *p*<0.001.

The total variance at the EA level was 2.040 in the empty model (Model 1). Model building began with the EA-level rationing and SES measures, and we proceeded to iteratively test combinations of covariates and first-order interaction terms until arriving at the most parsimonious model shown in Table 3. After adjusting for covariates, the full model without interactions (Model 2) minimized EA variance at 1.691, a decrease of 17.1%. Substantial variance remains, and this result suggests that living in a particular neighborhood strongly influenced the likelihood that a woman relied on sachet drinking water.

Table 3 gives the restricted pseudo-likelihood estimates for determinants of sachet use. We observed no support for our second hypothesis that neighborhood rationing was driving sachet consumption; the rationing variable was not statistically significant, though it did contribute to minimizing higher-level variance in the model. There is, as seen in Table 1, mixed support for our first hypothesis. At the individual level, lower self-reported overall health was strongly associated with sachet consumption: we observed a trend of increasing odds of sachet use for each category of overall health, with women reporting fair or poor health being significantly more likely to have used sachets than women reporting excellent health after adjusting for covariates (*p*<0.001). Age was also statistically significantly associated with sachet use, and the negative coefficient suggests that women were slightly less likely to use sachet water as their primary water source for each additional year of age, after controlling for covariates (*p*<0.01). The EA-level SES measure was also statistically significant; women in the lower class SES quartile were more likely (*p*<0.01) to use sachets than women in the upper class quartile, and we observed a similar trend of increasing odds of sachet use for each progressively lower SES quartile as seen with the *overall health* measure. Women without access to a sewer connection were also more likely to rely on sachets, but this difference was not statistically significant. These results affirm the profile of the “younger, less-well-off” sachet consumer, except that the wealth score was again positively associated with sachet use: women were more likely to use sachets (*p*<0.01) for each unit-increase in wealth score, which translated to a 20-percentile change when the score is centered on zero. This implies a massive relative increase in wealth score to produce a modest increase in the odds of sachet consumption, but it does indicate that even after controlling for covariates–particularly SES quartile–relative wealth still did impact the use of sachet water. Evidence of a possible interaction between SES and wealth appears in Table 4 where we stratified the mean wealth score for sachet users and non-users by SES quartile. Only the lower class quartile yielded a statistically significant difference in wealth score, as sachet users scored higher than expected (−0.15 vs. −0.58)–a difference that translated to the 54^th^ vs. 35^th^ percentile–and higher than sachet users in the lower-middle quartile (−0.34).

**Table 4:** Differences in wealth score between sachet users and non-users stratified by SES quartile.

	Sachet Water		Other Water Source	
Characteristic	Freq.	mean (95% CI)	Freq.	mean (95% CI)
*Individual characteristics (*%, *n = 2,814)*	190	6.8%	2,624	93.2%
Wealth score ∼		0.11 (−0.02–0.24)		−0.01 (−0.05–0.03)
* By SES quartile*				
Lower class *	78	−0.15 (−0.31–0.02)	707	−0.58 (−0.63– −0.53)
Lower middle class	36	−0.34 (−0.56– −0.11)	605	−0.27 (−0.34– −.021)
Upper middle class	36	0.35 (0.04–0.66)	681	0.16 (0.08–0.23)
Higher class	40	0.79 (0.47–1.11)	631	0.71 (0.62–0.79)

∼ *p*<0.10;

* *p*<0.001.

Note: *p-*values are from Welch *F* test of equality of means to account for variance heterogeneity.

Models 3–5 in Table 3 summarize the beta coefficients and standard errors for three additional models that introduce interaction terms to the full model. Model 3 contains cross-level interactions between rationing and proportion of vegetated land cover, and between rationing and SES, but these relationships were not statistically significant. We added cross-level interactions in Model 4 to test if rationing and SES interacted with overall health and wealth score. Only the interaction between lowest-quartile SES and wealth score–the relationship depicted in Table 4–was statistically significantly associated with higher sachet use (*p*<0.01). While most of the interaction terms in Model 4 were not statistically significant, their presence in the model rendered the beta for *days of rationing* marginally significant (*p*<0.10) and *proportion of vegetated land cover* statistically significant (*p*<0.01), thus lending initial, though weak, support to hypothesis 2 that increased rationing was associated with increased sachet use. At the individual level, living in the lower class SES quartile, worse overall health, and younger age were all associated with higher sachet use just as in Model 2. There was no strong evidence in Model 4 for hypothesis 3, which posits that the interaction between higher rationing and lower SES quartile–or any proxy for lower SES such as worse overall health or lower wealth–was associated with higher sachet use.

Model 5 contains additional individual-level interactions between overall health and wealth score, and between overall health and age. The interaction between overall health and age was statistically significant: for each additional year of age, women with fair or poor health (*p*<0.01), or very good health (*p*<0.10), were less likely to use sachets than women with excellent health, and the beta coefficients revealed a stronger effect for age than in Models 2–4. Just as in Models 3 and 4, worse overall health, living in a lower class EA, higher rationing, and a higher proportion of vegetated land cover were predictive of sachet use.

Models 3–5 present a more nuanced picture of the predictors of sachet use than Model 2, though the general conclusion is similar: younger, poorer women (or women living in poorer EAs) in neighborhoods experiencing higher rationing were most likely to rely on sachet water. The sole factor with a relationship contrary to hypothesis 1 was the significance of vegetated land cover at the EA level: a higher percentage of vegetation–which was statistically significantly associated with higher-SES quartiles in Table 5–was also predictive of sachet use. Previous research has shown a higher proportion of vegetated land cover to be associated with higher-status areas, not poverty [22], so this relationship may be an artifact of the transition in sachet consumption from higher to lower socioeconomic classes. The modeling of interaction terms in Table 3 lent weak support for hypothesis 2, as days of rationing persists as a marginally significant neighborhood-level influence on sachet use. Models 3 and 4 explain a slightly greater proportion of EA-level variance than Model 2, but the use of individual interaction terms in Model 5 does not improve overall results. There is no strong support for hypothesis 3; there are no significant interactions between rationing and any SES indicator.

**Table 5:** Enumeration area (EA) differences in rationing, proportion of vegetated land cover, and sachet use stratified by SES quartile.

		Days of Rationing	Vegetation (%)*	Sachet Use (%)
SES Quartile of EA	*N*	mean (SE)	mean (SE)	mean (SE)
Lower class	48	2.18 (0.26)	5.03 (0.91)	12.06 (3.00)
Lower middle class	50	2.12 (0.25)	8.03 (1.27)	6.69 (1.95)
Upper middle class	53	1.75 (0.20)	15.17 (1.85)	5.62 (2.13)
Higher class	44	1.84 (0.23)	25.06 (2.42)	8.53 (2.73)
Total	195	1.97 (0.12)	13.24 (1.01)	8.14 (1.23)

* *p*<0.001 from Welch *F* test of equality of means to account for variance heterogeneity.

## Discussion and Conclusion

Over the last decade sachet water has risen from relative obscurity to become an important source of drinking water throughout West Africa. Previous research has explored sachet water in select neighborhoods of Accra [8], [23] but this is the first study to analyze sachet water consumption in a West African metropolis using a socioeconomic and geographic cross-sectional approach. This study uses data collected in 2008–2009 to examine intra-urban variability of drinking water selection in Accra, and factors that influence these choices. We observe evidence of our first hypothesis, as sachet consumers tend to be younger, of poorer overall health, and of lower socioeconomic means, and yet those living in the economically poorest areas may be slightly better-off than their immediate neighbors. We also observe limited support for our second hypothesis that water rationing at the neighborhood scale positively influences sachet use, though rationing may be less important than individual-level factors. A smaller neighborhood unit was more appropriate for modeling sachet use, though there is still a considerable unexplained neighborhood effect. These neighborhood-level findings are consistent with recent reports from Accra [8]. The analysis does not support our third hypothesis regarding an interaction between rationing and lower socioeconomic status.

Two other recent population-based surveys, Measure DHS’s Ghana Demographic and Health Survey (DHS) and the Harvard/SDSU Housing and Welfare Study of Accra (HAWS), each report higher rates of sachet consumption than those reported in the WHSA-II [8], [23]. The last two Ghana DHS indicate that between 2003 and 2008 the percentage of urban households using sachet water as the primary water source increased from 6% to 37% nationally [24]. In Greater Accra, 87% of sachet-using households were in the top wealth quintile in the 2003 survey; by 2008 this rate had fallen to 71% and sachet use started trickling down into the middle and lower quintiles, but with scant evidence that sachets were a phenomenon of poverty [23], [24]. The WHSA-II data were collected between October 2008 and March 2009 and yield only 10% (78/785) of lowest-SES quartile participants relying on sachets; this survey seems to have been administered amidst a widespread transition to sachet water by lower-income populations. The HAWS data, collected between September 2009 and March 2010 exclusively in Accra slum neighborhoods, report 50% of households depending on sachet water with lowest-income residents as the most likely consumers [8].

Both 2008 and 2010 were especially bad years for water service delivery as power outages and construction projects at both of Accra’s water treatment plants led to multiple-week service interruptions in many neighborhoods. Sachet water was already a fairly ubiquitous product in Accra in 2008. It is plausible that sachet water’s higher unit price prevented it from becoming a *primary* drinking water source for many low-income communities until severe piped water shortages finally made sachets a necessity, even if temporarily. This study’s finding that sachet users in the lowest SES quartile score higher on a wealth index than sachet users in the lower-middle quartile underscores the reality that, in lieu of abject poverty, sachets may still be a discretionary, but increasingly attractive, choice to younger, poorer urban residents.

There are also several geographical discrepancies between the sachet patterns observed in the WHSA-II and those seen in the 2008 DHS and HAWS. In particular, the WHSA-II reports no sachet use among 270 women interviewed in the coastal Ga neighborhoods of Gbegbeyise, Chorkor, Korle Gonno, and Jamestown in western Accra. The DHS and HAWS data sets report significant sachet reliance in these and other neighborhoods where WHSA-II reports no sachet use. These coastal Ga neighborhoods in particular are known to have built much of their own water infrastructure (in a mix of legal and pirated networks), and the lack of maintenance by GUWL may increase the frequency of service disruptions. Given the geographic and temporal heterogeneity of water service in Accra, it is possible that some neighborhoods were interviewed in atypically water-abundant or water-scare periods, thus potentially biasing responses during any of these three surveys. It is also possible that there may have been some inadvertent bias in how the WHSA-II question about primary drinking source was framed to participants, despite all three surveys using the same DHS format. This mismatch between surveys may account for the lack of significant effects among neighborhood-level measures in this study, particularly days of rationing as observed in 2009–2010 [8]. More broadly speaking, it also highlights the difficulty of accuracy assessment for primary data collected in a developing urban setting. These survey discrepancies may soon be reconciled by the forthcoming release of 2010 Ghana Census results, as the 2010 questionnaire split the traditional household water question into separate items for drinking water sources and all other household water sources. The 2010 census may provide the most detailed picture yet of Ghana’s evolving drinking water trends.

This analysis is limited by the cross-sectional nature of the WHSA-II data. Despite compiling a broad array of women’s health outcomes, the WHSA-II did not measure diarrhea prevalence or other outcomes that might specifically be attributable to drinking water quality, thus it is difficult to assess the strong association between sachet use and overall health. Higher self-reported overall health has previously been positively associated with higher socioeconomic amenities in Accra [16], [25], [26], and ill-health has long been linked to chronic poverty [27], so the overall health measure can reasonably be interpreted as a SES proxy. Future research should directly target the relationship between sachet consumption and health outcomes suggested in the HAWS project [8].

Urban drinking water patterns in the developing world remain understudied, as urban areas are generally presumed to have sufficient piped water coverage. Accra is emblematic of many developing cities where this assumption is no longer valid [3], [9], [28]–[30]. Piped water access is lagging in urban sub-Saharan Africa [3], and residents are increasingly turning to privately vended water such as sachets. Problems with the unsustainable amount of plastic waste generated by sachet water consumption have been reported elsewhere [8], [23], [31], and have resulted in increased local media coverage of municipal water delivery issues faced by Ghana’s Ministry of Water Resources, Works and Housing. Sachet water continues to spread throughout West Africa, and many cities are likely experiencing similar transitions in drinking water patterns without a complete understanding of potential health effects or an appreciation for the environmental sanitation consequences of all the plastic waste. Global safe water and sanitation goals face many challenges [32], but in Ghana improvements are primarily constrained by financial resources; the capital investments required for an adequate water supply and sanitation infrastructure are estimated at $1.3 billion for the rehabilitation and expansion of urban water infrastructure alone [33]. Local governments and non-governmental organizations interested in alternative sustainable water provision would be well-served to understand the demographic appeal of sachet water, as well as the geographic and economic realities that turn citizens into sachet customers.
